# Comparison of Hydrophilic Properties of Titanium and Zirconia Dental Implants’ Surfaces

**DOI:** 10.3390/ma18081724

**Published:** 2025-04-09

**Authors:** Tadej Čivljak, Ticijana Ban, Vlatko Kopić, Valentina Petrović, Luka Morelato, Marko Vuletić, Dragana Gabrić

**Affiliations:** 1 Department of Oral Surgery, Dental Polyclinic Zagreb, 10000 Zagreb, Croatia; civljaktadej@gmail.com; 2 Center for Advanced Laser Techniques, Institute of Physics, 10000 Zagreb, Croatia; ticijana@ifs.hr; 3 Department of Maxillofacial and Oral Surgery, University Hospital Center Osijek, 31000 Osijek, Croatia; kopicv@gmail.com; 4 Faculty of Medicine Osijek, Josip Juraj Strossmayer University of Osijek, 31000 Osijek, Croatia; 5 Private Dental Practice, 10000 Zagreb, Croatia; dr.valentinapetrovic@gmail.com; 6 Private Dental Practice, 52440 Poreč, Croatia; morelatoluka@gmail.com; 7 Department of Oral Surgery, University of Zagreb School of Dental Medicine, 10000 Zagreb, Croatia; mvuletic@sfzg.hr; 8 University Hospital Centre Zagreb, 10000 Zagreb, Croatia

**Keywords:** dental implant, hydrophilicity, wettability, contact angle, osseointegration

## Abstract

One of the key factors influencing osseointegration is the hydrophilicity of the surface of dental implants; high hydrophilicity is more advantageous than low hydrophilicity. This study aimed to compare the hydrophilic properties of titanium and zirconia implants from different manufacturers. An in vitro analysis was conducted on 15 implants—13 titanium and 2 zirconia—each featuring distinct compositions and surface treatments. Their hydrophilicity was assessed using the contact angle method, where a drop of saline solution was pipetted onto the apical part of the implant. For each implant, 30 contact angle measurements were taken at three different surface wetting time intervals. The contact angle is defined as the internal angle between the tangent to the surface of the liquid and the surface at the point of tangency; a smaller angle means a higher hydrophilicity. The results show that titanium implants from BTI UniCa, Nobel TiUltra, and Straumann Roxolid SLActive—which are classified as premium implants—exhibited the highest hydrophilicity. In contrast, zirconia implants demonstrated significantly lower hydrophilicity. Within this group, the Nobel Pearl implant exhibited smaller contact angles than the Bredent WhiteSKY implant. Our findings confirm that high-quality titanium implants show superior hydrophilicity, potentially improving clinical outcomes by accelerating healing and facilitating immediate loading protocols, but this could only be proven with an in vivo animal study. Conversely, the relatively lower hydrophilicity of zirconia implants highlights the need for continued advancements in zirconia composition and surface modification to optimize their osseointegration potential.

## 1. Introduction

Several factors are widely known to be essential for the predictable osseointegration of dental implants: the biocompatible material of the implant, a minimally traumatic procedure to minimize tissue damage, placement in a satisfactory position surrounded by healthy bone, and the immobility of the implant during the healing phase. Osseointegration is also influenced by numerous local and systemic factors. Local factors affecting osseointegration include the operator’s technique, the implant design, the surface treatment, and the hydrophilicity of the dental implant, also known as wettability. Systemic factors that may influence the outcome of implant–prosthetic therapy include diseases or harmful habits that impair healing, such as diabetes, hyperlipidemia, and smoking, or treatments such as radiation in the head and neck area [1,2,3].

Regarding implant material, studies have shown that hydrophilic implants lead to better clinical outcomes than hydrophobic ones during the early phase of bone healing, with better bone-to-implant contact (BIC) and primary stability, indicating their suitability for critical situations such as a low bone density and immediate loading [4,5,6,7,8]. Hydrophilic dental implants promote adhesion and faster bone cell spreading when placed intraosseously. Increasing the hydrophilicity of the implant surface enhances the interaction between the surface and the surrounding biological environment [3,9]. Nanotubular surfaces with superhydrophilic characteristics have shown increased osteoblastic activity and significant expression of key genes associated with bone formation compared with hydrophobic surfaces. Also, it has been proven that the implant surface damage decreases the hydrophilicity of dental implants. Hence, manufacturers and current studies suggest wet storage until surgical use to prevent damage to the hydrophilic properties of implants [10,11,12].

Hydrophilicity is generally defined as a material’s affinity for water. Surface hydrophilicity and hydrophobicity are wetting properties resulting from the interaction between a liquid and a solid surface. The hydrophilicity of a surface is most commonly measured using the contact angle method, also known as the wetting angle. When a droplet of liquid is placed on the surface of a material, the attractive forces between liquid molecules create surface tension, resulting in droplets assuming a dome shape. The contact angle is defined as the internal angle between the tangent to the surface of the liquid and the surface at the point of tangency. A contact angle measurement is most often performed by depositing a droplet of liquid on the surface of a material. The material must be fixed when using this method, and the internal angle between the droplet and the surface is measured after the droplet makes contact with the flat surface. When the measured contact angle is less than 90°, the surface is hydrophilic. If the contact angle is less than 10°, the surface is superhydrophilic. In contrast, if the contact angle exceeds 90°, the surface is hydrophobic, and an angle greater than 150° indicates a superhydrophobic surface [13,14].

The internal contact angle decreases over time due to the evaporation of the saline water droplet, causing a decrease in the volume of the droplet and changing its shape from semi-circular to flattened. For this reason, the average contact angle was not calculated across all measurements; only the average contact angle was calculated at the same time point. The thermodynamics of water evaporation on a hydrophobic surface are complex but have been studied extensively [15].

The hydrophilicity of a dental implant’s surface is determined based on its surface treatment (i.e., the chemical composition of the surface). The surface of dental implants can be smooth or rough; roughness increases the implant’s surface area, accelerating osseointegration and achieving additional mechanical bonding with the environment. Techniques for modifying implant surfaces can be divided into additive and ablative techniques [16]. Ablative techniques remove material from the surface of the implant, thus creating pores. These methods include sandblasting, etching, anodizing, ball milling, and laser treatments. Various additive techniques are used to obtain a porous surface on titanium and titanium alloys through the addition of particles, with plasma spraying being a widely used technique [17].

Numerous titanium and zirconia dental implants from many manufacturers are available on the market, including relatively inexpensive ones that meet tested clinical conditions for use and more expensive “premium” implants. Manufacturers invest significant financial resources into clinical testing of the latter and meeting strict standards. Many manufacturers produce zirconia dental implants that are derived from zirconium dioxide (ZrO_2_) and which are suitable for anterior use due to their better esthetics. A study by Comisso et al. [18] showed that the esthetic and clinical advantages, as well as the survival and success rates, of zirconia implants are generally better than those of titanium implants. Therefore, we decided to include zirconia implants with titanium implants in our study examining hydrophilicity.

Due to the recent focus on hydrophilicity and the lack of data about the hydrophilicity of dental implant surfaces and comparisons between different manufacturers in the literature, this study compared the hydrophilicity of dental implants from various manufacturers, focusing on different surface treatments, such as trusted sandblasted implants with larger or smaller grit sizes, acid-etched treatments at higher or lower temperatures, and coatings with different layers. Additionally, the hydrophilicities of titanium and zirconia implant surfaces were compared. The null hypothesis was that there would be no statistical difference in surface hydrophilicity between different manufacturers and differently treated surfaces and no significant difference between titanium and zirconia implants. The alternative hypothesis was that the premium implants would have a higher surface hydrophilicity than low-cost implants.

## 2. Materials and Methods

This study was conducted in the Department of Oral Surgery at the Clinical Hospital Center Zagreb and the Centre for Advanced Laser Techniques at the Institute of Physics in Zagreb, Croatia.

The in vitro study examined 15 dental implants—13 titanium and 2 zirconia—from various systems and with different surfaces, as shown in Table 1.

**Figure 1:**
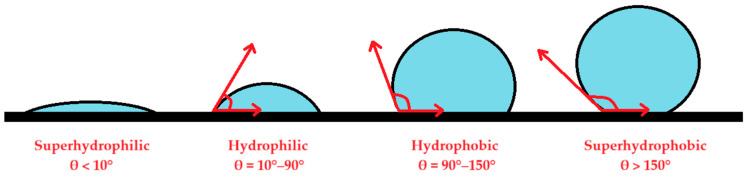
Schematic views of superhydrophilic, hydrophilic, hydrophobic, and superhydrophobic surfaces of solid materials.

Hydrophilicity was measured using the contact angle method, in which a droplet of constant volume of saline solution was pipetted onto the apical part of an implant. The contact angle is formed at the interface where a liquid droplet meets a solid surface. The angle quantifies the surface’s wettability, indicating the balance of adhesive forces between the liquid and soflid and cohesive forces within the liquid. The contact angle method was chosen for this study because the threads on the dental implants precluded using the immersion method, which would have required lateral hydrophilicity measurements. Different cases of surface wetting are illustrated in Figure 1.

Due to the specific conical shape of dental implants with threads, their hydrophilicity could only be measured by releasing a droplet onto the apical part of each implant. Given the diameter of the apical part, the droplet could not exceed a volume of 1 microL. This factor limited this study to implants with a larger diameter. Additionally, longer implants were chosen for easier fixation in the holder. The implants had diameters ranging from 4.5 mm to 7 mm and lengths between 12 mm and 17 mm. Implants with a pointed, overly narrow, or excessively convex apical part were excluded from the study, as they had no other flat surface, and measurements could not be taken using the selected technique.

**Figure 2:**
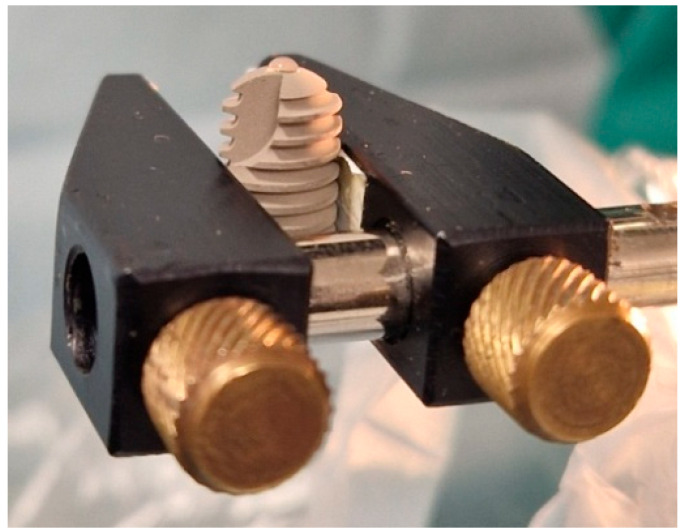
A dental implant (Ankylos^®^) in a holder with a droplet of physiological saline on the apical part.

The measurement process was performed by removing the implant from its box and holding it by its holder or by the cervical part of the implant if it lacked a holder while wearing sterile gloves, without touching the apical part of the implant where the measurements were taken. The implant was then fixed to a holder in the Optical System for Contact Angle Measurement and Contour Analysis, model OCA 15EC (DataPhysics Instruments GmbH, Filderstadt, Germany) as shown in Figure 2.

**Figure 3:**
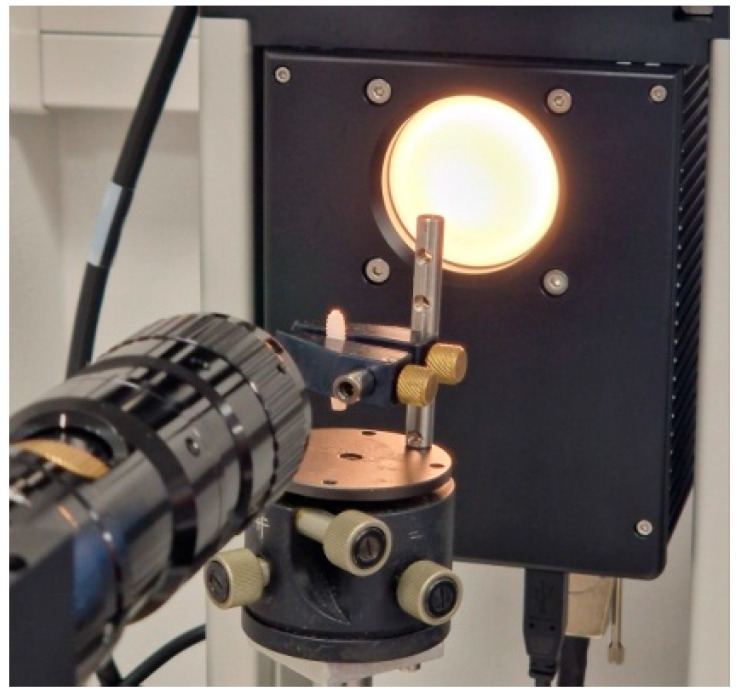
A dental implant (Bredent whiteSKY) fixed on the holder and set in the Optical System for Contact Angle Measurement and Contour Analysis.

This device consists of a platform that can be moved in all directions and fixed with a magnetic base, a fast 6.5-fold zoom lens with manual focus, and an adjustable observation angle, combined with a camera with a USB 3 interface and a manual dosing system (single direct dosing system, SD-DM), as shown in Figure 3.

**Figure 4:**
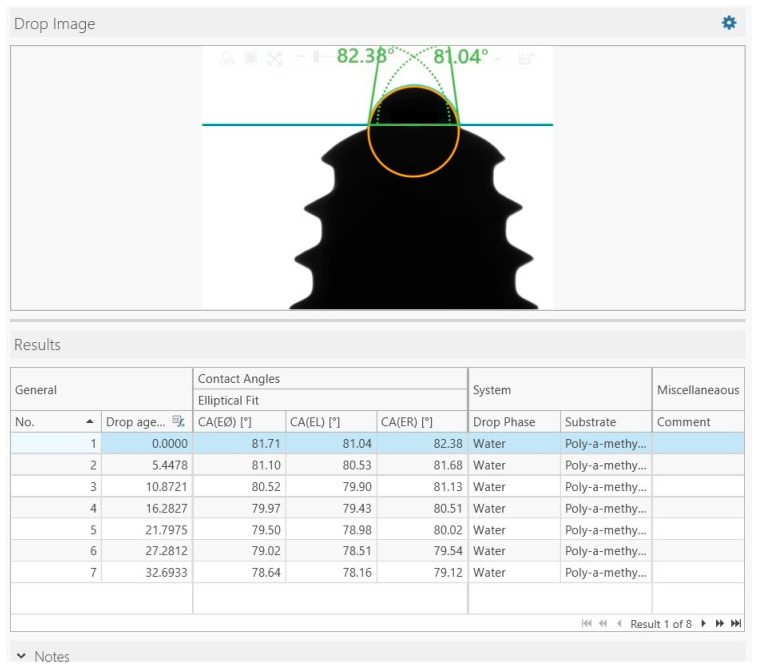
Example of contact angle measurement and calculation for one of the dental implants (Bredent whiteSKY).

The Optical System for Contact Angle Measurement and Contour Analysis was connected to a computer, and parameters were set in the dpi Measure Analyse Xplore program (DataPhysics Instruments GmbH, Filderstadt, Germany). The parameters were ambient phase—air; drop phase—water; continuous evaluation—5″; stop after 30″; and inner contact angle (i.e., the inner angle between the droplet of saline solution and the implant surface). It was not possible to use the manual dosing system integrated into the OCA 15EC because the syringe diameter was too large, leading to droplets with excessive volume/diameter that overflowed over the apical part of the implant. Therefore, a Gilson Pipetman P20 micropipette (Gilson S.A.S., Villiers-le-bel, France) with Gilson Diamond^®^ Tipack pipette tips, volumes 0.1–10 microL (Gilson S.A.S., Villiers-le-bel, France), was used for droplet dosing instead. A total of 1 microL of saline solution was pipetted from the bottle, and this volume of liquid was released onto the apical part of the dental implant from a distance of 2 mm without touching the implant surface. Immediately after contact was made between the droplet and implant surface, the contact angle measurement was started in the dpi Measure Analyse Xplore program at three time points, as shown in Figure 4.

A total of 30 contact angle measurements were taken for each implant: 10 at 5 s, 10 at 15 s, and 10 at 30 s after the droplet was dispensed onto the implant surface. The procedure began by dispensing a droplet onto the implant surface, followed by recording the contact angle at 5, 15, and 30 s. The droplet was then blown off the implant’s surface with a Cleaning Air Blowing Ball (Nedis, Hertogenbosch, The Netherlands) without touching the surface, and the surface was allowed to dry for approximately one minute before the second round of measurements commenced. Ten rounds of measurements were performed for each of the 15 implants.

Two implants, Straumann Roxolid^®^ SLActive^®^ and ICX-Active Liquid, were packed in boxes with liquid media (0.9% NaCl) to keep their surfaces intact before surgical use. Therefore, after fixing them on the measurement stand, their apical part was blown off and dried without touching the surface with the Cleaning Air Blowing Ball between each measurement.

### Statistical Analysis

The sample size was determined with an assumed significance level of α = 0.05 and a test power of 1 − β = 0.8, applying a non-parametric ANOVA test. To compare the 15 implant surfaces and detect a medium effect size (f = 0.25), this study included 450 measurements, with 10 measurements taken for each implant at three time points. The results of the measurements were described using the mean and standard deviation. All hypotheses were tested within a generalized linear model. For sample size calculation, the program G*Power version 3.0.10 (Heinrich-Heine-Universität Düsseldorf, Düsseldorf, Germany) was used. All hypotheses were tested with a significance level of α = 0.05.

## 3. Results

The surface hydrophilicities of 13 titanium and 2 dental zirconia implants from various manufacturers were investigated at three time points: 5, 15, and 30 s after a droplet was added to the implant surface.

The highest surface hydrophilicity amongst the titanium implants was achieved with BTI UniCa, Nobel TiUltra, and Straumann Roxolid SLActive, which are classified as premium implants. Ankylos’ hydrophilicity was closest to these three, while implants such as Alpha Bio MultiNeO, Astra, Dentium Implantium, GC Aadva, and ICX Active Liquid, could be classified into the medium-hydrophilicity category. Avinent, BTI Optima, Bredent BlueSKY, and PRAMA, showed the lowest surface hydrophilicities, with the latter being a borderline hydrophobic surface.

For BTI UniCA (Friedman test, *p* < 0.001) and Nobel TiUltra (Friedman test, *p* = 0.001), a significantly larger contact angle was observed after 5 s compared to after 15 and 30 s. After 5 s, significant differences were found in the contact angle between all types of implants (Kruskal–Wallis test, *p* < 0.001). For BTI Optima (Friedman test, *p* = 0.002), a significantly smaller contact angle was observed after 30 s compared with 5 and 15 s.

Significant differences were found between the implants after 5, 15, and 30 s (Kruskal–Wallis test, *p* < 0.001), as shown in Table 2.

In the zirconia implant group, significantly higher contact angles, indicating weaker hydrophilicity, were observed for the Bredent WhiteSKY implant compared with the Nobel Pearl at all time points (Mann–Whitney U test, *p* < 0.001).

When comparing the change in contact angle across measurement points within each implant type, significantly higher angles were observed after 5 s compared with 15 and 30 s and when comparing the angles that were measured after 15 s versus 30 s (Kruskal–Wallis test, *p* < 0.001), as shown in Table 3.

When comparing the hydrophilicities of zirconia and titanium implants from the same manufacturer (Nobel Pearl vs. Nobel TiUltra, Bredent WhiteSKY vs. Bredent BlueSKY), significantly higher contact angles—indicating weaker hydrophilicity—were found for the zirconia Nobel Pearl implant compared with the titanium Nobel TiUltra, and for the titanium Bredent blueSKY implant compared with the zirconia Bredent whiteSKY, as shown in Table 4 and Table 5.

When examining the sample distribution of the zirconia implants according to hydrophilicity, the Bredent WhiteSKY implant exhibited one hydrophobic sample at 5 s and one at 15 s, while the Nobel Pearl samples were hydrophilic at all three time points.

The present study also highlights the importance of wet storage, as evidenced by the superior hydrophilicity performance of the implants that were stored in liquid media.

## 4. Discussion

The null hypothesis was proven incorrect because there was a large difference in surface hydrophilicity between various titanium implants and between titanium and zirconia implants. The premium titanium implants demonstrated superior hydrophilicity, while the weaker hydrophilic properties of zirconia and some titanium implants highlight the need for continued innovation in surface treatments to optimize their performance. The smallest measured contact angles, which indicate the highest hydrophilicities amongst the titanium implants, were achieved by BTI UniCa, Nobel TiUltra, and Straumann Roxolid SLActive, which are classified as premium implants. In the zirconia implant group, significantly lower contact angles, indicating higher hydrophilicity, were observed for the Nobel Pearl at all time points compared with the Bredent WhiteSKY implant.

There are various studies in the literature that emphasize the importance of surface hydrophilicity in improving the osseointegration potential of dental implants and that ultrahydrophilic surfaces significantly contribute to early bone–implant contact (BIC) and primary stability, especially in critical clinical situations such as low bone density and immediate loading protocols [19,20,21]. The interaction between the implant surface and the biological environment significantly affects osseointegration; hydrophilic surfaces enhance this interaction by facilitating rapid protein adsorption and promoting osteoblastic activity [22,23,24]. Therefore, more hydrophilic implants may have better and faster osseointegration, but in vivo animal studies would be required to prove this.

The premium titanium implants demonstrated superior hydrophilicity, characterized by lower contact angles and rapid transition to ultrahydrophilic states. These properties support faster bone cell adhesion and proliferation, and accelerating healing.

In this study, the surface treatments achieving the highest hydrophilicities were BTI UniCa’s standard titanium surface modified with calcium ions [25]; Straumann SLActive’s surface, which is based on a large-grit sandblasting technique that generates macro-roughness on the titanium surface, followed by acid-etching, which superposes micro-roughness onto the surface [26]; and Nobel TiUltra’s anodized surface, with a gradual change in topography, which becomes moderately rough and porous towards the implant apex, where our measurements were taken, and which is also preserved with a protective oxide layer that dissolves upon contact with fluids [3,27]. The lowest hydrophilicities were those of the PRAMARF Sweden&Martina’s ZirTi body surface, which was sandblasted with zirconium and acid-etched with mineral acids [27], and Bredent blueSKY’s sandblasted and acid-etched surface treatment, the details of which are not described in the literature [28]. Rupp et al. [29] reported similar results, finding that the SLActive surface of various dental implants exhibited a 0-degree contact angle, indicating complete and rapid wettability. This superhydrophilic property is designed to promote faster osseointegration. Gittens et al. [14] reviewed the biological and clinical aspects of implants’ surface wettability, noting that hydrophilic surfaces enhance protein adsorption and cell adhesion, which are critical for early osseointegration. They also highlighted that surface modifications leading to increased hydrophilicity can improve clinical outcomes. Considering that the means of constructing implants with greater surface hydrophilicity and their advantages are known, it would be beneficial for other manufacturers to start treating the outer surfaces of implants similarly.

Kido et al. [30] investigated the effect of surface contaminants and hydrocarbon pellicles on the wettability of new titanium surfaces and titanium up to 90 days old stored under dark ambient conditions in a sealed package at 25 °C for up to 90 days. They found that titanium surfaces gradually become more hydrophobic due to the accumulation of organic contaminants, but treatments such as ultraviolet (UV) irradiation can restore superhydrophilicity. This finding highlights the importance of surface engineering and maintenance in preserving the desired hydrophilic properties of implants. Although there have been no specific studies on BTI’s UniCa and Nobel Biocare’s TiUltra implants, the consensus in the literature [31] supports the conclusion that advanced surface engineering techniques increase hydrophilicity—as seen with Straumann’s SLActive—and improve bone cell adhesion and proliferation, leading to accelerated healing and osseointegration. This study shows that implants stored in a moist state had excellent hydrophilicity; therefore, all manufacturers are advised to consider such a storage method to protect the implants from their manufacture to placement in the patient’s bone.

This research shows that, despite offering esthetic advantages, the zirconia implant Nobel Pearl exhibited weaker hydrophilic properties than its titanium counterpart, Nobel TiUltra. This situation was reversed for Bredent, where the zirconia implant showed greater hydrophilicity than the titanium one, but the difference was not as significant as with Nobel. The higher contact angles that were observed for both Bredent implants indicate a need for enhanced surface modification techniques to improve their wettability and, consequently, their osseointegration potential. Aragoneses et al. [32] concluded that titanium and zirconium implants have similar strength, hydrophilicity, and surface energy properties, but roughness causes a decrease in the hydrophilic character. In view of the above, it would be ideal if the implant was sufficiently rough for better osteointegration and sufficiently smooth for greater hydrophilicity.

Zirconia, which is commonly used in zirconia implants, exhibits larger contact angles than other restorative dental materials, suggesting lower hydrophilicity. This characteristic may influence the initial interactions between the implant surface and biological fluids, potentially affecting osseointegration [33]. In contrast, titanium implants often undergo surface modifications to enhance their hydrophilicity. For instance, anodization can increase the surface energy of titanium, resulting in lower contact angles and improved wettability. These hydrophilic surfaces have been associated with better cell attachment and accelerated healing processes. The dynamic nature of implant surfaces’ wettability is also noteworthy. Factors such as surface contamination can alter hydrophilicity over time, underscoring the importance of maintaining clean implant surfaces to preserve their beneficial properties [29]. Our research also confirmed that titanium implants have greater hydrophilicity than zirconium implants. Recent advancements in zirconia implant surface engineering, including laser-induced nanopatterning, offer promising solutions to these limitations [4,34,35]. The clinical advantages of hydrophilic implant surfaces extend beyond osseointegration. Improved wettability has been associated with enhanced primary stability, reduced healing times, and suitability for immediate loading protocols [29,36,37].

In the present study, the premium implants with ultrahydrophilic surfaces demonstrated superior performance, making them advantageous in challenging scenarios such as poor bone quality or compromised systemic conditions. These findings support the growing trend toward using hydrophilic implants in contemporary implantology. For instance, hydrophilic surfaces may improve outcomes in patients with systemic diabetes or osteoporosis, where delayed healing is often observed.

This study also highlights the importance of maintaining hydrophilic properties through wet storage, as evidenced by the superior performance of the implants stored in liquid media. This practice prevents contamination and the degradation of surface properties, ensuring optimal clinical outcomes [21,38]. Such innovations in implant storage and handling reflect a broader emphasis on preserving the functional integrity of implant surfaces, from manufacturing to placement.

It is worth emphasizing that surface wettability is a dynamic property that is influenced by various factors, including surface contamination and aging. Kido et al. [30] noted that the hydrophilicity of implant surfaces could diminish over time due to the adsorption of hydrocarbons from the environment, leading to increased contact angles and reduced wettability. The findings of Gittens et al. [14] underscore the necessity for real-time evaluations to monitor these changes and implement measures to maintain optimal surface characteristics for successful osseointegration. These authors also discussed the dynamic nature of implant surface wettability, highlighting that surface modifications and treatments can alter contact angles over time. Rupp et al. [39] highlighted that understanding these temporal changes is essential for predicting the biological performance of implants and ensuring long-term success. Real-time assessments of contact angles provide valuable insights into how surface properties evolve, enabling the development of strategies to preserve or enhance hydrophilicity throughout an implant’s lifespan. Our research shows that contact angles decrease, indicating greater surface hydrophilicity, over time, especially with premium dental implants such as BTI UniCa, which has proven to be the best regarding this.

The observed disparities between titanium and zirconia implants highlight the need for continued innovation in zirconia implant design. Advanced surface treatments, such as nanopatterning and chemical modifications, will enhance zirconia implants’ wettability and biological performance [40,41]. The development of bioactive zirconia surfaces that mimic the properties of natural bone is also worth exploring. Integrating hydrophilic zirconia implants into clinical practice will require rigorous testing to ensure their long-term durability and biocompatibility.

Despite its reliable methodology, this study was not without limitations. The in vitro nature of the hydrophilicity measurements may not fully replicate in vivo conditions, where factors such as blood viscosity and tissue interaction play crucial roles. Moreover, this study focused on the apical portion of the implants, which may not represent the entire implant surface’s hydrophilicity. Future research should address these limitations by incorporating in vivo studies and exploring the impact of hydrophilicity on long-term clinical outcomes. Such studies could also investigate the biological mechanisms underpinning the observed trends, including the molecular pathways that are activated by hydrophilic surfaces. Longitudinal studies assessing the long-term outcomes of hydrophilic implants in diverse patient populations will guide the development of next-generation implant systems.

## 5. Conclusions

The premium titanium implants with ultrahydrophilic surfaces tested in this study demonstrated superior wettability, while the weaker hydrophilic properties of zirconia and some titanium implants highlight the need for continued innovation in surface treatments to optimize their performance. The results underscore the benefits of advanced surface engineering and wet storage in maintaining optimal implant properties.

## Figures and Tables

**Table 1:** Dental implants included in this study.

Dental Implant	Material	Surface Treatment	Manufacturer	Dimensions (Diameter × Length)	Diameters of the Apical Part
Alpha Bio MultiNeO	titanium	sandblasted and acid-etched TiO_2_ protective layer	Alpha Bio Tec Ltd., Modi’in, Israel	5 × 13 mm	3.3 mm
Ankylos^®^	titanium	grit-blasted and high-temperature-etched	Dentsply Implants Manufacturing GmbH, Hanau, Germany	7 × 14 mm	5 mm
Astra Tech OsseoSpeed™ TX	titanium	grit-blasted (TiO blast) and acid-etched	Dentsply Implants Manufacturing GmbH, Hanau, Germany	5 × 17 mm	2.5 mm
Avinent	titanium	surface with included calcium and phosphorus (detailed information unavailable)	Avinent Implant System S.L., Santpedor, Barcelona, Spain	5 × 13 mm	3.5 mm
BTI UniCa	titanium	titanium surface modified with calcium ions (detailed information unavailable)	B.T.I. Biotechnology Institute S.L., Minano-Alava, Spain	5.5 × 11.5 mm	3.5 mm
BTI Optima	titanium	without calcium ions (detailed information unavailable)	B.T.I. Biotechnology Institute S.L., Minano-Alava, Spain	5.5 × 13 mm	3.5 mm
Bredent blueSKY	titanium	sandblasted and acid-etched	Bredent medical GmbH & Co., KG, Senden, Germany	4.5 × 14 mm	3 mm
Bredent whiteSKY	zirconia	sandblasted	Bredent medical GmbH & Co., KG, Senden, Germany	4.5 × 14 mm	3 mm
Dentium Implantatium	titanium	sandblasted with large grits and acid-etched	Dentium Co., Suwon, Republic of Korea	5 × 14 mm	3 mm
GC Implant Aadva^®^ Standard	titanium	sandblasting with high purity of alumina particles and acid etching	GCTech.Europe GmbH, Breckerfeld, Germany	5 × 12 mm	3 mm
ICX-Active Liquid Implant	titanium	(detailed information unavailable)	Medemtis Medical GmbH, Bad Neuenahr-Ahrweiler, Germany	4.8 × 15 mm	2.5 mm
Nobel Parallel™ CC TiUltra™	titanium	anodized and preserved with protective layer	Nobel Biocare AB, Göteborg, Sweden	5.5 × 15 mm	2 mm
Nobel Pearl™ Tapered WP	zirconia	ZARAFIL™ surface modified with sandblating and acid etching	Nobel Biocare AB, Göteborg, Sweden	5.5 × 12 mm	3 mm
PRAMARF Sweden&Martina	titanium	sandblasted with zirconia and acit-etched with mineral acids	Sweden&Martina S.p.A. Due Carrare—Padova, Italy	5 × 15 mm	2 mm
Straumann Roxolide^®^ SLActive^®^	titanium	large-grit sandblasted and acid-etched	Institute Straumann AG, Basel, Switzerland	4.8 × 12 mm	3.5 mm

**Table 2:** Measured contact angles after 5, 15, and 30 s for 13 titanium implants from different manufacturers.

		Median (IQR)						*p* * (Between Measurements)
		5 s	*p * ^†^	15 s	*p * ^†^	30 s	*p * ^†^	
Ankylos	(1)	37.1 (27.7–47.8)	**<0.001 ** ^║^	25.6 (0–39.8)	**<0.001** **	23.2 (0–36.8)	**<0.001 ** ^††^	0.08
Astra	(2)	55.4 (54.2–57.5)		54 (52.9–56.5)		53.4 (49.1–55.2)		0.16
BTI UniCA	(3)	18.8 (16.9–20.7)		0 (0–0)		0 (0–0)		**<0.001** ^‡^
Nobel TiUltra	(4)	24.4 (20.8–26.2)		15.1 (10.3–17.9)		0 (0–0)		**<0.001** ^§^
Straumann roxolid SLActive	(5)	21.8 (20.8–22.3)		19.8 (19.5–20.3)		19.8 (0–20)		**0.001** ^‡^
Alpha Bio MultiNeO	(6)	50.3 (47.8–54.1)		41.0 (38.5–49.3)		30.4 (28.2–42.5)		**<0.001** ^§^
Avinent	(7)	73.7 (73.4–74.8)		72.4 (71.5–73.1)		70.9 (70.3–71.8)		**<0.001** ^§^
BTI Optima	(8)	78.8 (78.3–79.6)		78.6 (77.7–79.6)		77.4 (76.9–77.9)		**0.002** ^§§^
Bredent blueSKY	(9)	96.8 (90.6–97.1)		91.2 (88.4–91.5)		88.9 (87.4–89.8)		**<0.001** ^§^
Dentium Implantium	(10)	58.3 (54.8–58.6)		56.7 (53.2–57.7)		55.4 (52.9–56.5)		**<0.001** ^§^
GC Aadva	(11)	50.1 (48.4–50.7)		49.1 (47.9–50.2)		47.1 (46.0–49.1)		**<0.001** ^§^
ICX Active Liquid	(12)	48.0 (47.6–49.5)		45.3 (43.4–46.1)		40.2 (38.8–42.7)		**<0.001** ^§^
PRAMARF Sweden&Martina	(13)	87.5 (86.0–90.9)		86.8 (85.3–90.2)		85.7 (84.2–89.3)		**<0.001** ^§^

Abbreviations and notes: IQR—interquartile range; **bold** values indicate statistical significance. * *Friedman test* (post hoc Conover test)—between measurements. ^†^ *Kruskal–Wallis test* (post hoc Conover test)—between implants. ^‡^ *p* < 0.05; significant differences between (5 min) and (15, 30 min). ^§^ *p* < 0.05; significant differences among all three time points. ^§§^ *p* < 0.05; significant differences between (30 min) and (5, 15 min). ^║^ *p* < 0.05; all implant types showed significant differences except (2) vs. (10), (3) vs. (5), (4) vs. (5), (6) vs. (11), (9) vs. (13), and (11) vs. (12). ** *p* < 0.05; all implant types showed significant differences except (1) vs. (5), (2) vs. (10), (4) vs. (5), (6) vs. (12), and (9) vs. (13). ^††^ *p* < 0.05; all implant types showed significant differences except (2) vs. (10), (3) vs. (4), and (9) vs. (13).

**Table 3:** Differences in measured contact angles after 5, 15, and 30 s for zirconia implants.

	Median (IQR)			*p* *
	5 s	15 s	30 s	
Nobel Pearl	68.6 (67.5–70.04)	67.3 (66.4–68.5)	66.4 (65.3–67.1)	**<0.001** ^‡^
Bredent whiteSKY	82.6 (81.1–88.9)	81.5 (79.9–87.2)	80.5 (78.6–84.9)	**<0.001** ^‡^
Difference ^§^	14.4	14.6	14.5	
95% CI	11.8 do 19.6	12.2 do 19.7	12.2 do 19.6	
*p* ^†^ (between implant types)	**<0.001**	**<0.001**	**<0.001**	

IQR—interquartile range; **bold** values indicate statistical significance. * Friedman test (post hoc test Conover); ^†^ Mann–Whitney U test; ^§^ Hodges–Lehmann median difference. ^‡^ At *p* < 0.05, significant differences for (5 s) vs. (15, 30 s) and (15 s) vs. (30 s).

**Table 4:** Differences in measured contact angles after 5, 15, and 30 s between zirconia and titanium implants from the same manufacturer (Nobel Pearl vs. Nobel TiUltra).

	Median (IQR)			p *
	5 s	15 s	30 s	
Nobel Pearl	68.6 (67.5–70.04)	67.3 (66.4–68.5)	66.4 (65.3–67.1)	**<0.001** ^‡^
Nobel TiUltra	24.4 (20.8–26.2)	15.1 (10.3–17.9)	0 (0–0)	**<0.001** ^§^
Difference	43.9	52.5	65.8	
95% CI	41.4 do 46.8	48.2 do 56.8	64.8 do 67.1	
*p* ^†^ (between implant types)	**<0.001**	**<0.001**	**<0.001**	

IQR—interquartile range; **bold** values indicate statistical significance. * Friedman test (post hoc test Conover); ^†^ Mann–Whitney U test; ^§^ Hodges–Lehmann median difference. ^‡^ At *p* < 0.05, significant differences for (5 s) vs. (15, 30 s), (15 s) vs. (30 s) and between all three times.

**Table 5:** Differences in measured contact angles after 5, 15, and 30 s between zirconia and titanium implants from the same manufacturer (Bredent whiteSKY vs. Bredent blueSKY).

	Median (IQR)			p *
	5 s	15 s	30 s	
Bredent whiteSKY	82.6 (81.1–88.9)	81.5 (79.9–87.2)	80.5 (78.6–84.9)	**<0.001** ^‡^
Brednet blueSKY	96.8 (90.6–97.1)	91.2 (88.4–91.5)	88.9 (87.4–89.8)	**<0.001** ^‡^
^§^ Difference	−10.9	−9.03	−7.9	
95% CI	−14.6 to −6.8	−11.8 to −3.5	−10.5 to −2.6	
*p* ^†^ (between implant types)	**<0.001**	**0.001**	**0.001**	

IQR—interquartile range; **bold** values indicate statistical significance. * Friedman test (post hoc test Conover); ^†^ Mann–Whitney U test; ^§^ Hodges–Lehmann median difference. ^‡^ At *p* < 0.05, significant differences for (5 s) vs. (15, 30 s), (15 s) vs. (30 s) and between all three times.

## Data Availability

All data relevant to the study are included in the article.

## Abbreviations

The following abbreviations are used in this manuscript:

BIC	bone-to-implant contact;
ZrO_2_	zirconium dioxide;
BTI	Biotechnology Institute.
